# Establishment and development of national community-based collaborative innovation demonstration areas to achieve the control target of hepatitis B in China

**DOI:** 10.1186/s12879-019-4150-9

**Published:** 2019-07-12

**Authors:** Bing Ruan, Zhixin Yu, Shigui Yang, Kaijin Xu, Jingjing Ren, Jun Yao, Nanping Wu, Chengbo Yu, Min Deng, Tiansheng Xie, Ping Chen, Chencheng Wang, Yiping Li, Yanhong Zhao, Jifang Sheng, Yunde Hou, Zunyou Wu, Shuigao Jin, Yude Chen, Mengfeng Li, Fengcai Zhu, Hong Tang, Yuantao Hao, Xinghuo Pang, Lin Lu, Wen Yang, Zhengan Yuan, Aiqiang Xu, Zizhao Li, Mingjian Ni, Yongping Yan, Qiu Zhong, Lin Zhou, Guojian Li, Qun Meng, Jianping Hu, Hong Zhou, Guangyu Zhang, Dexin Li, Wei Jiang, Qing Li, Peixin Wu, Ruoqi Xing, Jinhui Gu, Di Gao, Lanjuan Li

**Affiliations:** 1 The State Key Laboratory for Diagnosis and Treatment of Infectious Diseases, Collaborative Innovation Center for Diagnosis and Treatment of Infectious Diseases, The First Affiliated Hospital of Zhejiang University, Zhejiang University School of Medicine, No.79 Qingchun Road, Hangzhou, China; 2 Zhejiang Provincial Center for Disease Control and Prevention, Hangzhou, China; 3 Chinese Center for Disease Control and Prevention, Beijing, China; 4 Peking University, Beijing, China; 5 Sun Yat-sen University, Guangzhou, China; 6 Jiangsu Provincial Center for Disease Control and Prevention, Nanjing, China; 7 Sichuan University, Chengdu, China; 8 Beijing Center for Disease Control and Prevention, Beijing, China; 9 Yunnan Provincial Center for Disease Control and Prevention, Kunming, China; 10 Sichuan Provincial Center for Disease Control and Prevention, Chengdu, China; 11 Shanghai Center for Disease Control and Prevention, Shanghai, China; 12 Shandong Provincial Center for Disease Control and Prevention, Jinan, China; 13 Henan Provincial Center for Disease Control and Prevention, Zhengzhou, China; 14 Xinjiang Provincial Center for Disease Control and Prevention, Urumchi, China; 15 The Forth Military Medical University, Xian, China; 16 The Center of Tuberculosis control of Guangdong Province, Guangzhou, China; 17 Guangxi Provincial Center for Disease Control and Prevention, Nanning, China; 18 National Statistical Information Center of China, Beijing, China; 19 The 309th Hospital of PLA, Beijing, China; 20 The Digital Medical and Health Technology Research Institute of Zhejiang Province, Hangzhou, China; 21 National Health and Family Planning Commission of China, Beijing, China

**Keywords:** Community healthcare, Hepatitis B, Health policy

## Abstract

**Background:**

The major infectious diseases of hepatitis B has constituted an acute public health challenge in China. An effective and affordable HBV control model is urgently needed. A national project of Community-based Collaborative Innovation HBV (CCI-HBV) demonstration areas has optimized the existing community healthcare resources and obtained initial results in HBV control.

**Methods:**

Based on the existing community healthcare network, CCI-HBV project combined the community health management and health contract signing service for long-staying residents in hepatitis B screening. Moreover, HBV field research strategy was popularized in CCI-HBV areas. After screening, patients with seropositive results were enrolled in corresponding cohorts and received treatment at an early stage. And the uninfected people received medical supports including health education through new media, behavior intervention and HBV vaccinations. In this process, a cloud-based National Information Platform (NIP) was established to collect and store residents’ epidemiological data. In addition, a special quality control team was set up for CCI project.

**Results:**

After two rounds of screening, HBsAg positive rate dropped from 5.05% (with 5,173,003 people screened) to 4.57% (with 3,819,675 people screened), while the rate of new HBV infections was 0.28 per 100 person-years in the fixed cohorts of 2,584,322 people. The quality control team completed PPS sampling simultaneously and established the serum sample database with 2,800,000 serum samples for unified testing.

**Conclusions:**

CCI-HBV project has established a large-scale field research to conduct whole-population screening and intervention. We analyzed the HBsAg prevalence and new infection rate of HBV in the fixed population for the epidemic trend and intervention effect. The purpose of CCI-HBV project is to establish and evaluate a practical model of grid management and field strategy, to realize the new goal to control hepatitis B in China. To provide policymakers with a feasible model, our results are directly applicable.

**Trial registration:**

The project was funded by the Major Projects of Science Research for the 11th and 12th five-year plans of China, entitled “The prevention and control of AIDS, viral hepatitis and other major infectious diseases”, Grant Nos. 2009ZX10004901, 2011ZX10004901, 2013ZX10004904, 2014ZX10004007 and 2014ZX10004008.

## Background

The infectious diseases of hepatitis B presents a severe threat to human health worldwide, and have caused severe problem in China, both financially and socially. In 2009, we initiated the Community-based Collaborative Innovation (CCI) project for the prevention and control of major infectious diseases including AIDS, hepatitis B and TB, which were in the list of Major Special Projects in the National Medium- and Long-term Program for Science and Technology Development (2006–2020) in China. The primary objective of CCI project was to explore the epidemiological baseline, influencing factors, and trend of three major diseases. And the long-range objective was to reduce morbidity and mortality of three diseases and to improve the capability of community healthcare to deal with infectious diseases in CCI areas by 2020.

HBV infection is wide-ranging with large differences between different countries. To understand the prevalence of HBV in China, Chinese National Health Department carried out national HBV sero-epidemiological investigations in 1979, 1992 and 2006. The results showed HBsAg carrier rates in 1–59 aged people was 8.75, 9.75 and 7.18%, respectively. [1–3] According to the result of 2006 screening, the estimated number of chronic HBV cases was 93 million in China. [4] In January 2006, Chinese National Health Department added HBV to National Disease Control and Prevention Plan List, indicating that HBV control was crucial for infectious diseases control in China. In general, the lack of HBV vaccinations in adults was responsible for HBV epidemic, indicating that national HBV vaccination for whole population (including infants and adults) holds greater promise for long-term treatment. [5, 6] Therefore, we conducted this CCI-HBV field research to explore a series of questions: the cause of HBV high prevalence in China, the different prevention strategies for different groups, the prognosis of HBV carriers, and the way to achieve periodic follow-up in local residents.

## Method

CCI project is a task-based research based on a nationwide system composed of two branches: the administrative branch, and the technical branch. Under the coordination of the Implementation Management Office of Special Projects for Infectious diseases in the National Health Department, the administration branch consists of a nation-, province-, and county-level executive leadership team, while the technical branch includes a design engineering group, a responsible expert group, and a field executive team. The two branches link together in the project by formulating an administrative evaluation index of local administrations. Thus, local government gains a unified plan, technical guidance and professional evaluation while the project obtains policy support, guaranteed funding, administrative cooperation and inspection.

### The field research of CCI project based on the existing urban and rural community health system

#### Community-based collaborative innovation (CCI) chain

**Fig. 1:**
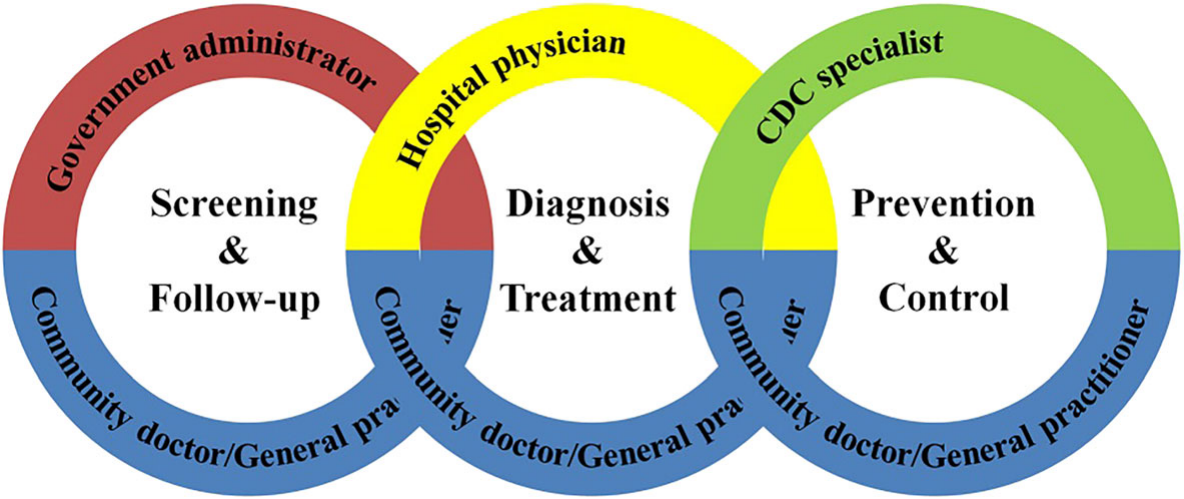
The construction of CCI chain

In combination with health administrators in county-level, physicians in county hospitals, and specialists in county centers for disease control (CDCs), CCI executive team in each county was on the existing community healthcare teams (i.e., community doctors and general practitioners). This emerged the basis of CCI chain, which is complementary in resource, discipline, profession and region (Fig. 1). In this way, CCI project provides long-staying residents with grid management, networking services, and digital information of diseases.

#### Community doctors/general practitioners in community healthcare clinics/centers provide residents with grid management and contract-signing service

The community healthcare clinic in urban residential areas and rural villages is the basic unit in community healthcare. The community doctor in clinics signed health contract with local long-stay residents (> 6 months/year) for health service (1 doctor:1000–1500 residents). The residents also gave their consent for confirming their health and vital data through Electronic Health Records (EHRs) review and further data analysis, including medical history, family history, vaccination history etc.

On this basis, one community healthcare center has been upgraded in accordance with several corresponding community clinics with physical examination equipment and computers. The general practitioners (GP) in center (teaming up with the community doctors in clinics) organized dynamic centralized HBV-related screening including abdominal ultrasonography and serological tests of HBsAg, HBeAg, ALT, anti-HCV, serum level of ALT and AFP. Then, they imported the results to EHRs through Hospital Information System (HIS) and Laboratory Information System (LIS) before submitting it to the regional information system, which links to the national information cloud platform (NIP). After the screening, community doctors would print out the results, send and explain results to the residents. To date, two rounds of health screening of HBV have been completed (2010–2012, 2013–2015), and the population was divided into uninfected group and infected group, to receive the corresponding prevention and intervention.

Several approaches were applied to maintain the operation of field research, including providing regular medical training for community healthcare staff and improving reward with Performance Reward Mechanism (KPI). The doctor’s fee-for-service solution of contract signing service kept doctor’ service quality. This also increased residents’ health compliance because of the flexibility of community service.

**Fig. 2:**
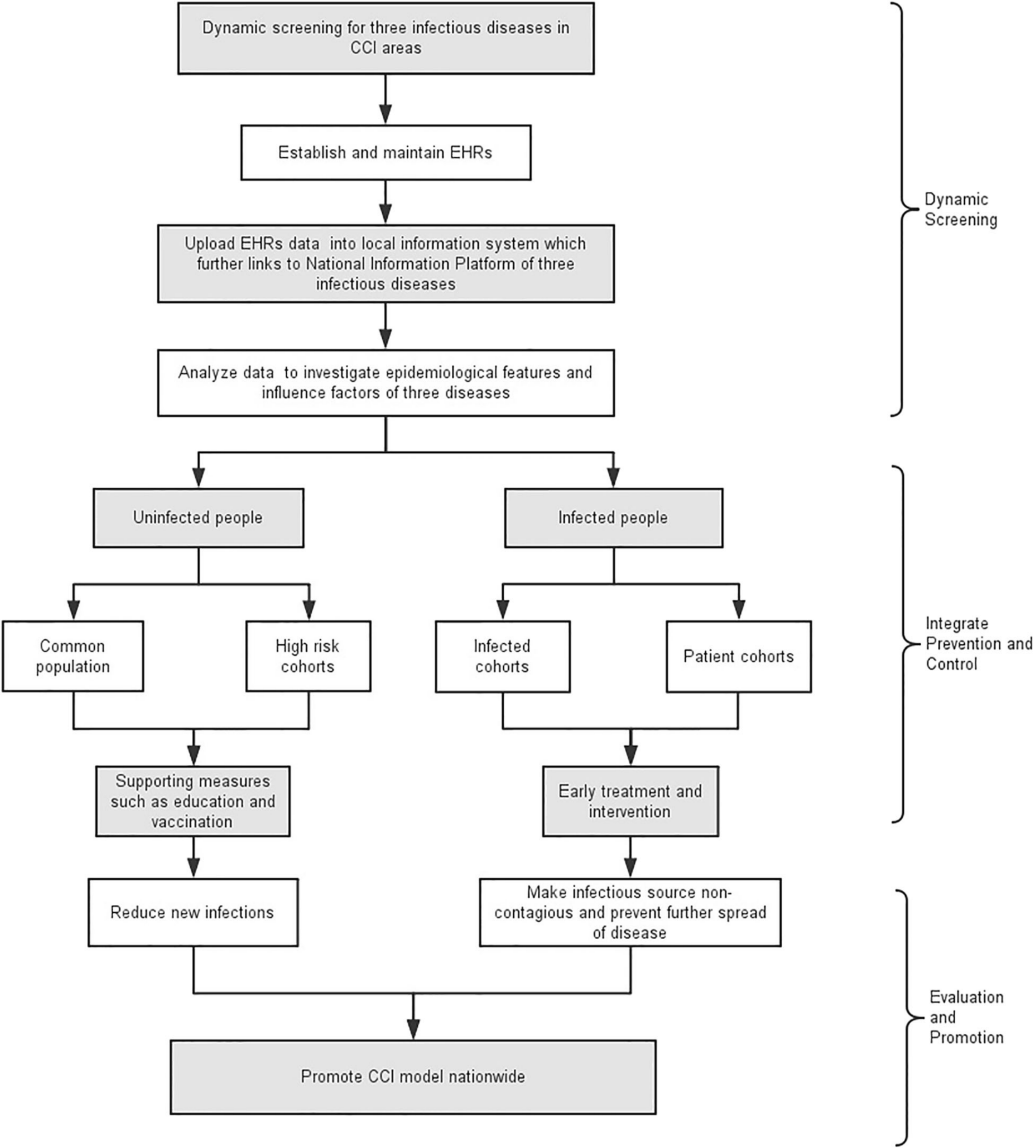
The Flowchart of community CCI Field Research

In short, the community healthcare team (composed of community doctors and GPs) plays the role of health gatekeeper for all residents in CCI areas. Figure 2 shows a flowchart of the field research.

#### Specialists in county hospital make the definite diagnosis and achieve two-way referral

Specialists in county hospitals made the definite diagnosis and recommended treatment for the suspected carriers/patients after screening. For patients in mild condition, the normalized treatment at early stage was recommended for good prognosis, and this group of patients would be back to community. Then, critical patients would be transferred to the higher-level hospital through green channel. This Two-way Referral also helped induct people into their corresponding cohorts and ensured that they would receive prompt treatment. In this way, CCI project saves medical resources, releases the pressure on tertiary hospitals and improves treatment efficiency.

### The strategy of CCI-HBV intervention

#### HBV: find all susceptible people, vaccinate all susceptible people, follow up all susceptible cases, and treat all patients (FVFT strategy)

This strategy offers free screening, free HBV vaccines for the uninfected group, and free antiviral treatment for long-staying residents in CCI-HBV areas. HBV-FVFT strategy occurs in four stages; diagnosis (the whole-population screening), prevention (building an immunological barrier in full-aged HBsAg negative people), normalized treatment (standard antiviral therapy), and follow-up (long-term follow-up to explore disease development and related factors). With timely treatment, HBV-infected individuals could avoid secondary complications such as hepatic failure, cirrhosis and HCC.

#### The community-based health education in CCI-HBV areas

Health people are the key for CCI intervention. To appeal more residents, a special WeChat Public Account is being designed to regularly deliver health information for all residents. Once the related science material is finished by doctors, it can be pushed to the residents’ smart phones synchronously. Meanwhile, local health people/patients can join self-management groups by interests and health conditions with the assistance of social workers, GPs and specialists. With peer supporting and interacting, people acquired medical knowledge with fun. In this way, the community-based health education in CCI-HBV areas improved the health education effect for individuals.

### Establishment and implementation of National Information Cloud Platform for infectious disease

As the first large-scale medical information platform in China, the national information cloud platform of infectious diseases was created jointly by the authority and a third-party (Zhejiang WaData Technology Co., Ltd). Due to the large physical distance and population, each CCI area collected its own data and uploaded onto the national platform respectively. Using a unified data submission solution, we created a safe medical data environment. We believe residents would get benefit from big-data analysis from individualized intervention to reliable diagnosis and treatment.

### Establishment and implementation of national quality control center for data verification

**Fig. 3:**
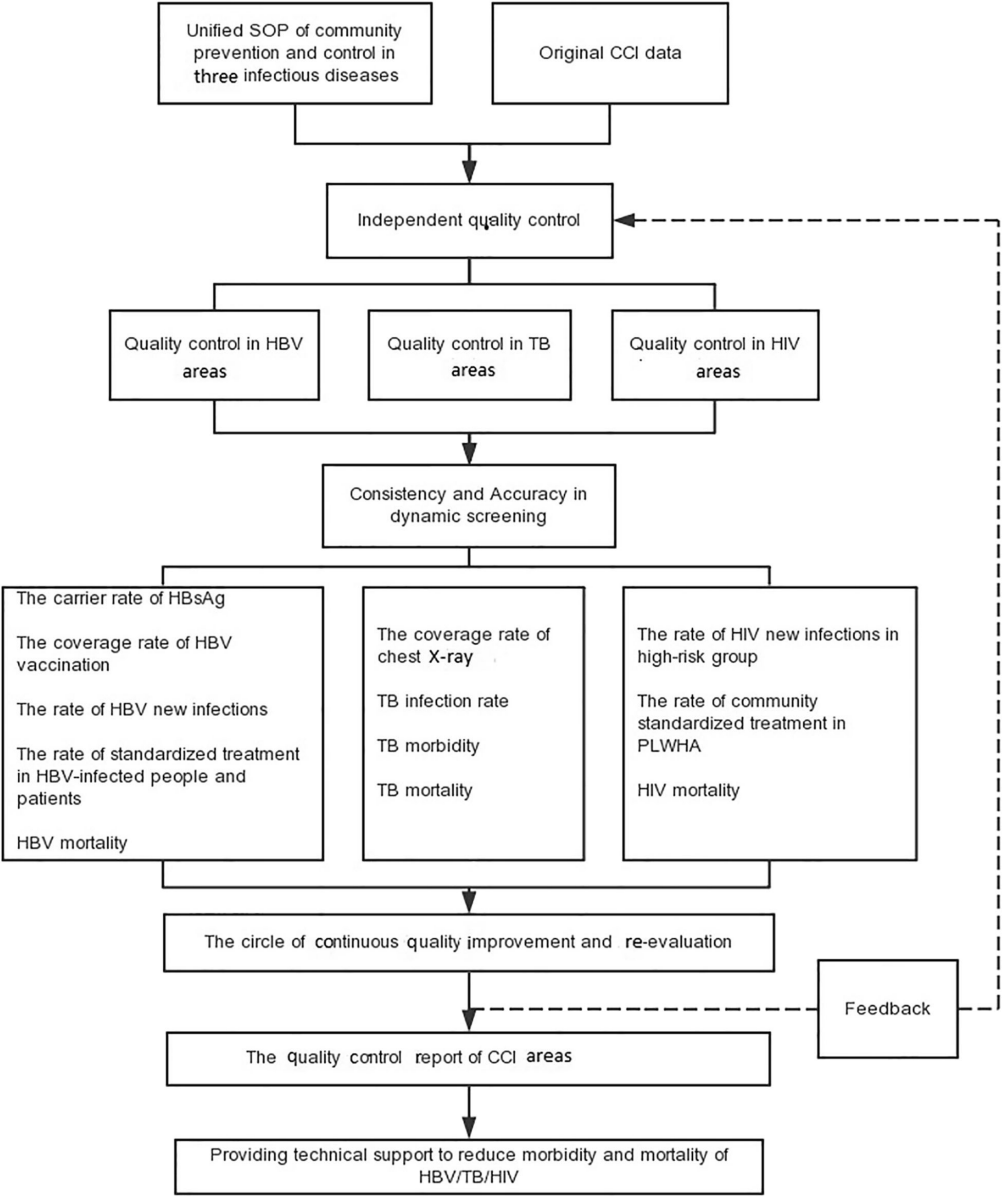
The Flowchart of Quality Control

The national quality control center consists of administrative staff in the implementation office of the Special Project for Infectious Diseases of Chinese Health Ministry, expert panels at different levels, and community quality controllers. While the expert panel is composed of experts in infectious diseases, epidemiologists, health statisticians, management experts, and information technology specialists. Figure 3 shows the quality control flowchart.

The following measures were taken to ensure a functional quality control operation.

1. Investigation of CCI areas

A basic information form was formulated by the national quality control center to gather information of CCI areas, including demographic information, health network information, equipment and professional training of community healthcare providers, the establishment of EHRs, and the screening data in local record, etc. The CCI province/city-level control team in HBV areas has established the serum sample bank using standardized test method and exclusive barcode tags.

(2) Sample surveys of the residents

The residential sampling survey in CCI areas was designed by the national quality control center, organized by each province/city quality control team, and specifically executed by each county/district quality control group and community quality controllers. The sampling method was the probability proportional to size sample (PPS sample) with 100 thousand people in CCI-HBV area in the second round of screening (2013–2015). The PPS specimens were sent to the headquarter in Hangzhou for unified quality control with quantitative reagent (Abbott Laboratories, Chicago, IL, USA). The residential sampling survey offered another perspective to review the application of Standard Operation Procedure (SOP) by verifying the consistency of information from the paper questionnaires, EHRs, to the national information cloud platform in PPS samples.

## Results

### Establishment and development of CCI-HBV areas

The national CCI areas were established in 2009 and came into operation in 2010. The selection of CCI areas was based on these factors: the disease burden, epidemiological characteristics, geographic and demographic features, and preliminary community healthcare foundations.

**Fig. 4:**
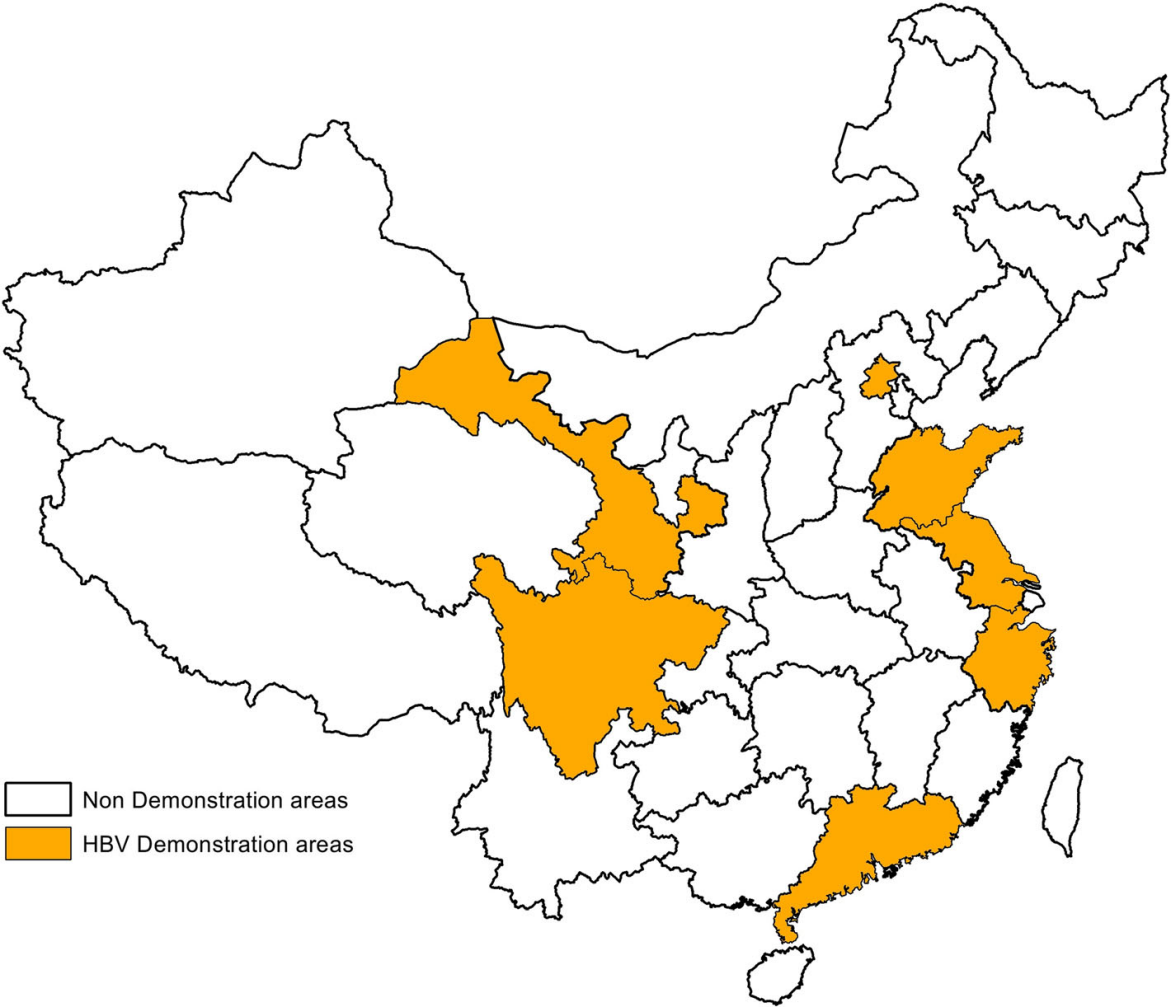
The Distribution of National CCI-HBV Dmonstration Areas

In total, 7 CCI-HBV areas were established and developed on different economic and epidemical conditions of infectious diseases by 2015, covering more than 12.00 million in HBV areas (Fig. 4). To date, CCI project is now relatively mature and effective. The entire development can be divided into three stages:

#### The 11th five-year plan period: 2009–2010

Based on the New Rural Cooperative Medical System which began in 2003 in China and covered all the rural residents by 2007, we started our project as below:

1. The dynamic community HBV screening was conduct of hepatitis B antigen-antibody, ALT, AFP, and abdominal ultrasound scan.
2. HBV cohorts were designed to assess the long-term effects of HBV intervention (see Table 1 for the list of HBV cohorts). By 2015, the populations of the cohorts were fixed. As a multi-center field research project, further result will be available at the end of third round screening of 2016–2018.

**Table 1:** The HBV-related cohorts in CCI-HBV areas

CCI area of diseases	Group	Cohort	Target capacity by 2020	Present capacity (2015)
HBV	Uninfected	HBV new infection rates in the general population cohort	> 1,000,000	1,360,000
		Adult HBV vaccination intervention cohort	> 500,000	639,799
	Infected	HBV carrier cohort	> 50,000	70,890
		Chronic HBV antiviral treatment cohort	> 30,000	34,083
		Hepatic cirrhosis antiviral treatment cohort	> 2000	2257

#### The 12th five-year plan period: 2011–2015

Based on the foundation set by the 11th five-year plan period, the following 5 years covered:

1. The Standard Operation Procedure (SOP) was proposed by the top design specialist team in 2011 to guarantee the quality of the project, concentrating on implementation specifications, and this SOP was registered in 2013 (registration number: country as the Word − 2013-L-00090505).
2. The data from the residents’ EHRs began to upload to the national information cloud platform which was completed in 2012 to assemble the large-scale data, laying the foundation for further analysis.
3. The quality control section was introduced in 2013 to assess the accuracy of the field research.

#### The 13th five-year plan period: 2016–2020

It is important to evaluate this study in the next stage. To evaluate the long-term effect of the cohorts, a follow-up of the fixed population in the community will be studied. Moreover, we plan to optimize the population structure of cohorts due to the population migrating.

### Initial screening for HBV in CCI areas

By December 31st of 2015, CCI project has completed two rounds of health screening for HBV from 2010 to 2012 (Round 1) and 2013–2015 (Round 2). According to NIP database, there were 5,173,003 people participated in HBV screening with 261,237 seropositive cases in 1st round, and 3,819,675 people with 174,559 seropositive cases in 2nd round of screening. The HBsAg seropositive rate in these seven HBV demonstration areas has dropped from 5.05% (95%CI: 5.03–5.07%) to 4.57%(95%CI: 4.55–4.59%) and the *P* value of the two screening results was less than 0.01 (Table 2). According to the data, the positive rate of HBsAg in the 2nd round of screening held significant difference from the first round. It could be said that the HBsAg prevalence in CCI-HBV areas was gradually decreasing. By compared two rounds of screening, the number of new HBV infection was identified as 21,997 in two rounds of 2,584,322 fixed people. With the follow-up interval of 3 years, the incidence rate in CCI-HBV areas was 0.28 (95%CI:0.27–0.29) per 100 person-years.

**Table 2:** The Screening for hepatitis B in CCI areas during 2010–2015

Year	Screening for hepatitis B				
	No. of individuals screened for HBsAg	No. of HBsAg positive individuals	HBsAg positive rate (%)	95% CI for HBsAg positive rate (%)	*P* value Round 1 to Round 2
Round 1 2010–2012	5,173,003	261,237	5.05	5.03–5.07	–
Round 1 2013–2015	3,819,675	174,559	4.57	4.55–4.59	< 0.01
Total	8,992,678	435,796	4.85	4.83–4.86	–

### Quality control of CCI-HBV areas

By the end of 2015, the national quality control center has completed two rounds of investigation synchronously with two rounds of screening, and the next round of investigation will be finished by the end of 2018. The feedback of quality control center verified the construction of CCI-HBV field research.

#### Investigation of health resources and detection capability

To date, CCI-HBV areas have equipped all demonstration areas with the relevant healthcare resources and detection capability (Table 3). The Microplate readers, biochemical analyzers and B ultrasound were available in CCI-HBV areas.

**Table 3:** Relevant health resources and detection capability in CCI-HBV areas

CCI Area of diseases	Covering population/million	No. of Community healthcare centers	No. of Community healthcare clinics	No. of Community responsibility doctors	HBV screening equipment and conditions		
					Biochemical analyzer	Microplate reader	B ultrasound machine
HBV	12.00	495	5655	12,992	480	184	675

To ensure the accuracy of residential identification, all CCI areas used personal ID numbers as unified tag. With unified serum sample transportation and barcode management, the serum sample database with 2.8 million samples has been established.

#### Investigation of the information consistency

A total of 80,000 PPS samples from HBV areas was collected separately in the end of the second round of screening. By comparing sample information with the NIP data, the agreement of consistent ID number was 98.7%, and the coincidence of HBsAg(+) was 96.1%. According to the quality control cross-sectional study, HBsAg(+) carrier rate in PPS samples was 5.44%. The quality control team has submitted self-evaluation report, which promotes CCI project with dynamic feedback and sustained development.

## Discussion

The epidemiological features of infectious diseases have been noticeably transformed in China. [7] HBV has proven to be a considerable challenge over the last few decades, [8] In 2014, the total number of reported cases of notifiable infectious diseases in China was 7,184,391, which included 935,702 HBV patients (360 deaths). [9]

Inspired by the experience of Framingham epidemiology program for cardiovascular disease in America which was initiated in the 1940s, CCI project tires to control and prevent the three major infectious diseases by improving operational capability in community healthcare units. [10–12] With the vast rural population, many residents obtain rudimentary treatment from community clinics which offer basic healthcare in China. Despite the lack of advanced equipment and highly-trained doctors, these clinics do have some advantages. Combined with the progress of Chinese New Medical Reform, CCI project promotes the regional concentration of medical resources. Instead of large tertiary hospitals in central areas, CCI focuses on integrating community healthcare centers and clinics which was a significant step taken after the SARS outbreak in 2002. [13–15] In this way, medical recourses can flow flexibly without causing hold-ups or waste. By 2015, the project had established an infectious disease prevention network consisting of more than 15,000 community clinics including 30,000 community responsibility doctors.

Many different methods have been implemented to deal with infectious diseases worldwide. Coates reported the effect of community-based voluntary counseling and testing for HIV in Africa. [16] Because the Brazillian government operates the largest program of free, highly active antiretroviral drug treatments in the world, there has been a surge in resistance to many antiviral drugs. [17] ZAMSTAR was a community-based randomized trial carried out in 24 communities in Zambia, to explore the prevalence of confirmed pulmonary TB in adults before and after intervention. [18] Compared with other studies, CCI project holds some features.

In 1992, a national HBV vaccination program was initiated in China and was aimed at all infants (1992 Program). The coverage vaccination rate of 1992 program ranged from 91.7 to 99.9%. [19] To further reduce the prevalence of HBV, a new vaccination program was implemented in CCI-HBV areas in 2011 (2011 Program), aimed at all healthy adults (HBsAb seronegative) and infants. As part of HBV-FTVT Strategy, 2011 program was different from 1992 program in many aspects including that the 10 μg recombinant hepatitis B vaccine was used to achieve immunization effect in CCI-HBV areas. [20] Together, the 1992 and 2011 Programs reduced HBsAg carrier rate in < 15 years-old in Zhejiang province to less than 1%. [19] The 2011 Program confirmed that universal vaccination acted as a protection by creating an immune barrier. [21] In harnessing the power of existing community healthcare programs, CCI areas have achieved long-term follow-up of HBV infections to verify the transformation of HBV carriers (the new infection rate, etc) and apply standard antiviral therapies to reduce patient mortality. These measures led to the HBV mortality in Zhejiang province decreased from 1.54% (2010) to 0.98% (2015). According to the screening data, the second round of HBsAg prevalence has decreased compared with the first round with significant difference. This also proves the effectiveness of CCI comprehensive intervention. In addition, the study of HBV new infection transformation is ongoing.

With the development of digital medicine, multiple medical data sources are now available, covering medical system data (HIS/LIS/RIS/PACS/EMR), health behavior data (continuously updated EHRs and personal portable devices), medical tests and medical records, etc. All these data sources constitute a large-scale database, which forms a relatively realistic picture of disease prevalence and cannot be ascertained by artificial calculation. For this reason, the NIP cloud computing was selected for the project data processing task. Now the national information cloud platform is available to all CCI communities and it’s under continual development with data uploading.

Quality control is vital for the epidemiological field research, which could help researchers to discover bias in the data collection process. By developing a quality control element in this project, we review the application of SOP in the field, to ensure the consistency of data from various areas to the national information cloud platform. In CCI quality control investigation, we found the population mobility has influenced the screening, follow-up and intervention, which would be the priority to be improved in the future.

A cross-sectional study showed low awareness of hepatitis B in rural China. [22] and a public health education program in CCI areas is urgently needed. With the help of community doctors and local social workers, we organized various self-management groups for both the uninfected people and infected people, to provide various activities with well-directed medical education in community. Meanwhile, a health education Wechat public account for smart phones has being popularized, because the accessibility of new media contributes to the dissemination of information on public health issues, especially for younger groups. [23] Thus, CCI-HBV improved people’s compliance and understanding, and contributed to the adoption of the grading treatment service in China.

## Conclusions

In summary, the large-scale CCI-HBV project is an innovative example to prevent and control hepatitis B in China. The study proved the effectiveness of CCI strategy and provide policymakers with a feasible reference object. In brief, CCI project is a reliable national intervention, which may take several years to prove itself in reducing the morbidity and mortality of hepatitis B in China.

## Data Availability

The datasets used and/or analyzed during the current study are available from the corresponding author on reasonable request.

## Abbreviations

CCI: Community-based Collaborative Innovation
DR: Digital Radiography
EHR: Electronic Health Record
EMR: Electronic Medical Record
FVFT Strategy: The strategy of CCI-HBV control, the acronyms of Find all susceptible persons, Vaccinate all susceptible persons, Follow up all susceptible persons, and Treat all patients
GP: General practitioner
HBV: Hepatitis B Virus
HIS: Hospital Information System
KPI: Key Performance Indicator
LIS: Laboratory Information System
NIP: National Information Cloud Platform
PACS: Picture Archiving and Communicating System
PPS Sampling: Probability-Proportional-to-Size Sampling
RIS: Radiology Information System
SARS: Sever Acute Respiratory Syndrome
SOP: Standard Operation Procedure
